# Iatrogenic esophageal and tracheal perforation with tracheoesophageal fistula following emergency intubation

**DOI:** 10.1002/ccr3.3552

**Published:** 2020-11-20

**Authors:** Akram Alkrekshi, Hazim Bukamur

**Affiliations:** ^1^ Department of Internal Medicine The MetroHealth System Campus of Case Western Reserve University Cleveland OH USA; ^2^ Department of Pulmonary and Critical Care Medicine Texas Tech University Health Science Centre Lubbock TX USA

**Keywords:** esophageal cancer, iatrogenic, tracheoesophageal fistula

## Abstract

Iatrogenic tracheoesophageal fistula (TEF) through direct penetration of esophageal and tracheal walls is exceedingly rare. Body tissues sealing around the tube may result in delayed development of respiratory complications and diagnosis. Pneumomediastinum and pneumothorax may be absent. Maintaining the airway through TEF until tracheostomy resulted in a satisfactory outcome.

## Graphical abstract

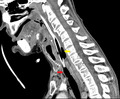

A 52‐year‐old female with a history of tongue squamous carcinoma treated with surgery and radiotherapy 6 years prior. A recent workup for dysphagia revealed an ulcerated friable mass at the postcricoid region that extended 8.5 cm distally. Flexible bronchoscopy showed posterior mid‐tracheal wall asymmetry. Biopsies confirmed cervical esophageal squamous carcinoma (T2N2M0). The patient presented with hematemesis complicated by asphyxiation and cardiopulmonary arrest. She was resuscitated with a return of circulation within 4 minutes. Intubation was difficult; bougie and video laryngoscopy were used to place a size‐6 endotracheal tube (ETT). Two days later, the patient developed acute respiratory failure. Computed tomography imaging showed bilateral consolidation and an abnormally placed ETT that traversed from the esophagus to the trachea resulting in tracheoesophageal fistula (TEF). There was no pneumomediastinum or pneumothorax (Figures 1, 2, 3, 4). Antibiotics started, and enteral feeding was held. Three days later, gastrostomy‐tube placement, tracheostomy, and ETT removal in the operating theater were done. Radiotherapy and chemotherapy followed with resultant remission.

**Figure 1:**
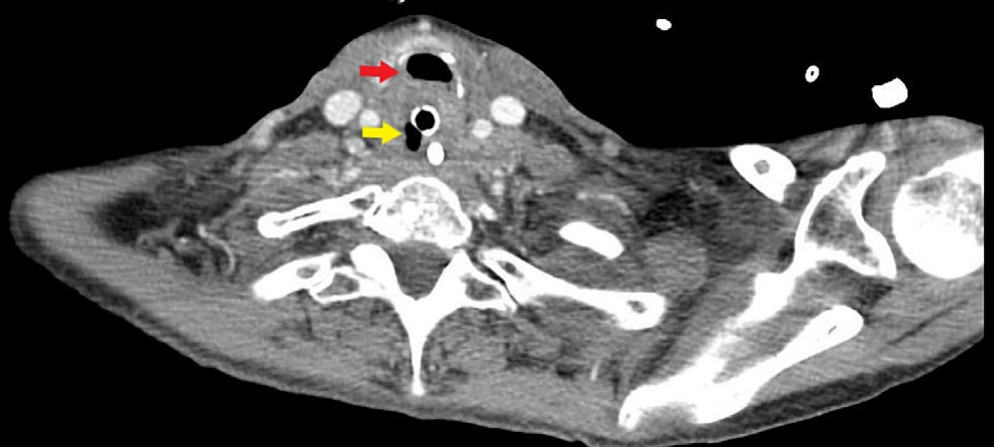
Endotracheal tube traversing the esophagus (yellow arrow). Trachea (red arrow)

**Figure 2:**
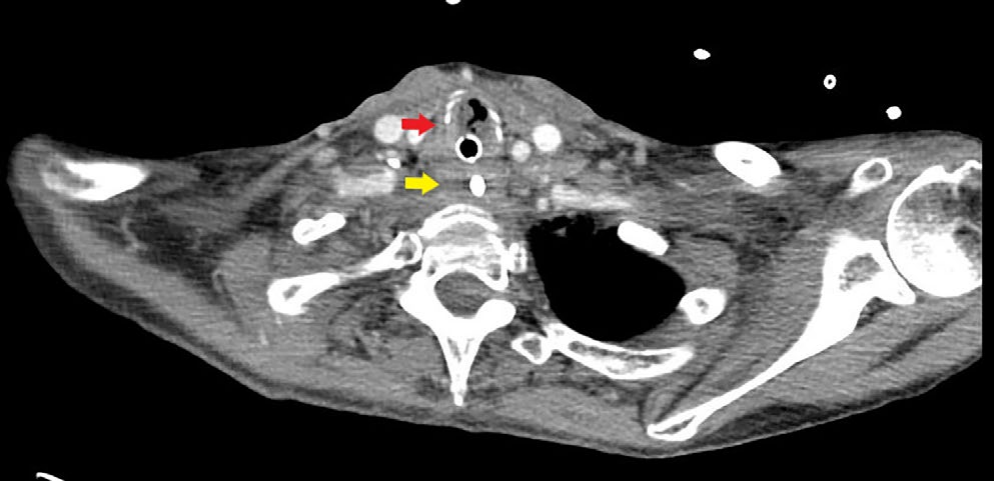
Endotracheal tube traversing the trachea (red arrow). Esophagus (yellow arrow)

**Figure 3:**
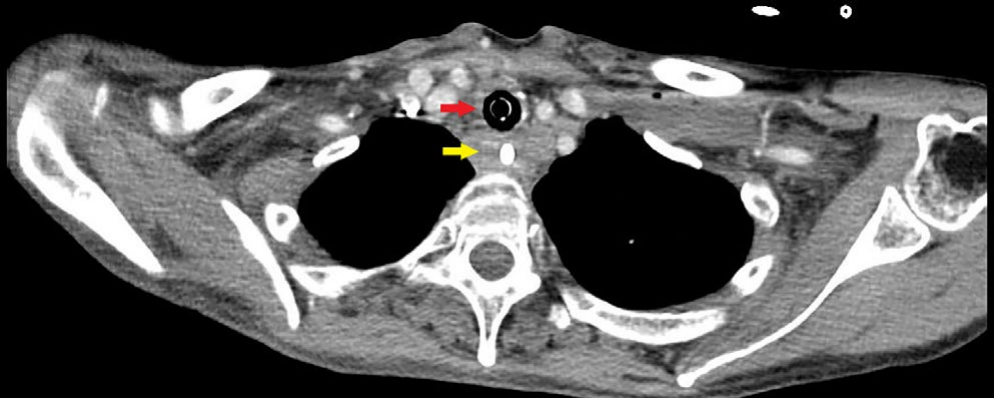
Endotracheal tube in the trachea (red arrow). Esophagus (yellow arrow)

**Figure 4:**
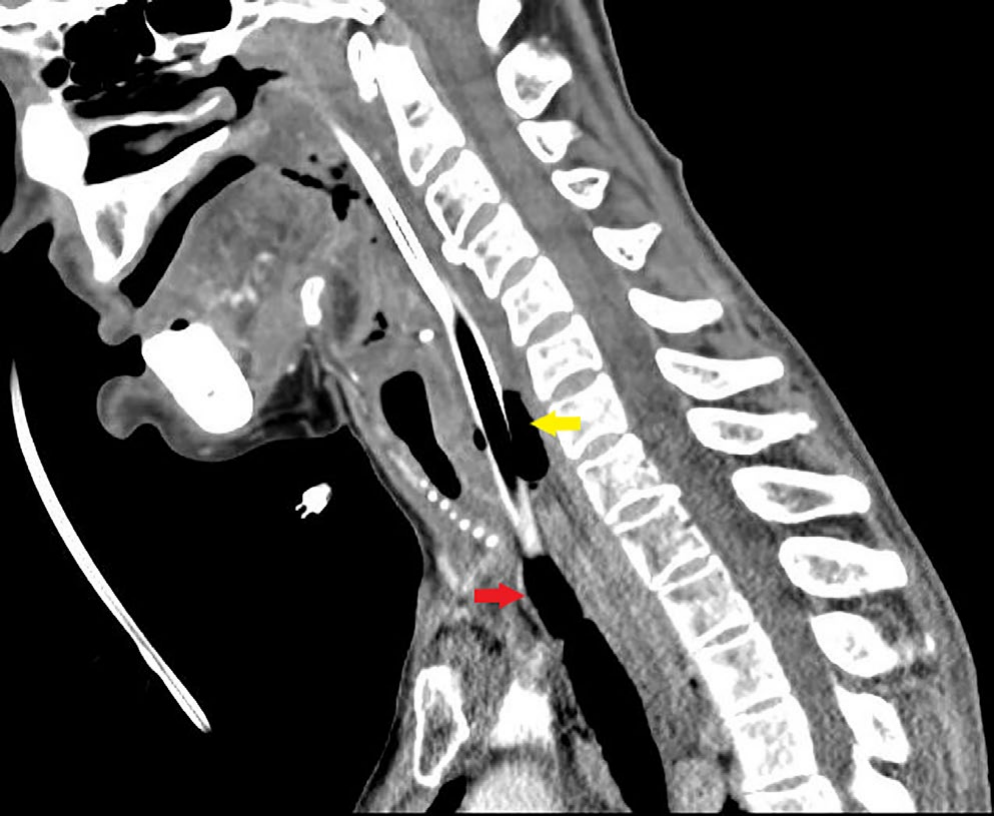
Endotracheal tube traversing the esophagus (yellow arrow) into the trachea (red arrow)

TEF is rarely iatrogenic and usually due to posterior tracheal wall erosion from pressure by an overinflated endotracheal cuff.^1^ This case is unusual as TEF was due to an errantly placed ETT. Maintaining the airway through TEF until tracheostomy and supportive measures resulted in a satisfactory outcome.

## CONFLICT OF INTEREST

We have no conflict of interest to disclose.

## AUTHOR CONTRIBUTIONS

Both authors have contributed significantly to draft preparation and manuscript editing.

## Data Availability

Data available on request from corresponding author.
